# Are School-Based Programs Effective in Tackling Childhood Obesity in Europe? A Systematic Review

**DOI:** 10.3390/nu18121916

**Published:** 2026-06-12

**Authors:** Cíntia Carneiro Gomes, Christos Triantafyllou, Joao Breda

**Affiliations:** WHO Athens Quality of Care and Patient Safety Office, Ploutarchou 3, 10675 Athens, Greece; cintia.gomes@who.int (C.C.G.); rodriguesdasilvabred@who.int (J.B.)

**Keywords:** childhood obesity, programs, diet, physical activity, Europe

## Abstract

**Background:** Childhood obesity remains a major public health challenge worldwide, with increasing prevalence across Europe. Schools represent an important setting for promoting healthy lifestyles through physical activity and nutrition-related interventions. This systematic review aimed to evaluate the effectiveness of school-based interventions promoting physical activity and healthy eating behaviours among children and adolescents aged 6–18 years in European countries. **Methods:** A systematic literature review was conducted using PubMed and Scopus. Studies were eligible if they were conducted in school settings, targeted children and adolescents aged 6–18 years, were implemented in European countries, had a minimum duration of nine months, and assessed anthropometric and/or behavioural outcomes related to obesity prevention. Methodological quality was assessed using the Effective Public Health Practice Project (EPHPP) Quality Assessment Tool. **Results:** Sixteen studies conducted across nine European countries met the inclusion criteria. Intervention duration ranged from nine months to five years, and most studies employed multicomponent approaches combining physical activity promotion, nutrition education, environmental modifications, and parental involvement. Seven studies were rated as strong quality, six as moderate quality, and three as weak quality. Among the fourteen studies assessing BMI or other anthropometric outcomes, eleven (78.6%) reported statistically significant improvements in at least one obesity-related measure, including BMI, BMI z-score, waist circumference, waist-to-height ratio, body fat percentage, or overweight/obesity prevalence. Evidence regarding physical activity and nutrition-related outcomes was more heterogeneous, although several studies reported improvements in dietary behaviours, nutrition knowledge, sedentary behaviour, and physical activity levels. Positive anthropometric effects were more commonly observed in interventions lasting at least one academic year and in multicomponent programmes. Some studies also reported differential effects according to sex and parental educational background. **Conclusions:** The findings of this review suggest that long-term, multicomponent school-based interventions can contribute to improving obesity-related anthropometric outcomes among children and adolescents in European countries. However, evidence regarding sustained changes in physical activity and dietary behaviours remains less consistent. Future research should focus on identifying the most effective intervention components and strategies for achieving long-term behavioural change across diverse populations and educational contexts.

## 1. Introduction

The World Health Organization (WHO) has identified childhood overweight and obesity as one of the most pressing public health challenges of the 21st century. The prevalence of childhood obesity has increased substantially worldwide, including across Europe, with important implications for population health and healthcare systems [1,2]. Children and adolescents with overweight or obesity are more likely to remain obese in adulthood and are at increased risk of developing non-communicable diseases such as type 2 diabetes, cardiovascular disease, and certain cancers at an earlier age [1,2]. The development of obesity is multifactorial, resulting from a complex interaction of behavioral, environmental, social, biological, and economic determinants [3]. Given the substantial amount of time children spend at school, the school setting has been widely recognized as a strategic environment for promoting healthy lifestyles and preventing obesity [4]. Schools can influence health behaviors through nutrition education, physical education programs, healthy food environments, and broader health-promoting policies [5]. Furthermore, in many European countries, children consume at least one meal during the school day, while food choices may also be influenced by the availability of snacks and beverages through school shops, cafeterias, and vending machines [6]. Consequently, school-based interventions have the potential to reach large populations of children and adolescents and contribute to sustainable improvements in dietary habits and physical activity levels. Previous research has demonstrated that school-based physical activity interventions can improve activity levels and contribute to the prevention of excessive weight gain among children and adolescents [7,8]. Similarly, interventions combining physical activity promotion with nutrition education have been associated with greater improvements in obesity-related outcomes, including body mass index (BMI), compared with single-component approaches [9]. Several systematic reviews have reported promising effects of school-based interventions on healthy lifestyle behaviors and obesity prevention [9,10,11,12,13,14]. In addition to their health benefits, improvements in physical activity and dietary behaviors have also been linked to enhanced cognitive function and academic performance [15]. Despite the growing body of evidence, findings across studies remain heterogeneous regarding intervention components, duration, implementation strategies, and effectiveness. Moreover, an updated synthesis focusing specifically on school-based interventions implemented within European countries is warranted, given the diversity of educational systems, sociocultural contexts, and public health policies across the region. Therefore, the present systematic review aims to evaluate the effectiveness of school-based interventions designed to promote physical activity and healthy eating habits among children and adolescents aged 6–18 years in European countries, assessing both behavioral outcomes (physical activity and dietary behaviors) and anthropometric outcomes related to obesity, including BMI and other measures of body composition.

## 2. Materials and Methods

### 2.1. Literature Search and Study Selection

This systematic review was conducted and reported in accordance with the Preferred Reporting Items for Systematic Reviews and Meta-Analyses (PRISMA) guidelines [16]. A systematic search of school-based physical activity and nutrition interventions aimed at addressing childhood obesity was conducted through electronic searches of scientific articles published between May 2010 and January 2026 in the PubMed and Scopus databases. The search strategy included the following keywords: (pediatric obesity OR childhood obesity) AND (exercise OR physical activity OR school program) AND (diet school program* OR school food program* OR school feeding program* OR school eating program*) AND Europe*. * After removing duplicate records, the remaining studies were screened in two stages. First, titles and abstracts were reviewed, and clearly irrelevant papers were excluded. Second, full-text articles were assessed in detail to identify studies that met the predefined inclusion criteria. This systematic review was not registered, and no separate review protocol was prepared.

### 2.2. Inclusion Criteria

Studies were included if they:

- Employed an intervention study design (e.g., randomized controlled trial, cluster-randomized trial, quasi-experimental study, controlled intervention study, or other longitudinal intervention design) and evaluated the effectiveness of a school-based programme promoting physical activity, healthy eating behaviours, nutrition education, or a combination of these approaches;
- Reported effects on obesity-related anthropometric outcomes (e.g., BMI, BMI z-score, body weight, waist circumference) and/or behavioral outcomes targeted by the intervention, including physical activity levels, dietary habits, nutrition-related behaviors, sedentary behavior, or other health-related lifestyle behaviors;
- Targeted children and adolescents aged 6 to 18 years attending primary and secondary school respectively;
- Were conducted at the school setting;
- Included a school-based intervention aimed at promoting physical activity, healthy eating behaviors, nutrition education, or a combination of these approaches, and had a duration of at least nine months;
- Were conducted in a population without health issues such as without eating disorders, dyslipidemia, mental or physical disabilities, diabetes, anemia, etc.
- Were not systematic reviews, reviews, meta-analysis, and editorials.

### 2.3. Data Extraction and Risk of Bias

After duplicate removal, two reviewers independently screened titles and abstracts for eligibility. Potentially relevant studies underwent full-text assessment by the same reviewers according to the predefined inclusion and exclusion criteria. Any disagreements were resolved through discussion and consensus. Data extraction was performed independently by two reviewers using a standardized data extraction form. Information extracted included study design, country, participant characteristics, intervention type, intervention components, duration, outcome measures, data collection methods, and main findings. Data extracted from the included studies were summarized in tabular form and synthesized narratively. Given the substantial heterogeneity across studies in terms of intervention characteristics, study designs, duration, outcome measures, and assessment methods, a quantitative meta-analysis was not feasible. Therefore, the findings were grouped and presented according to the main outcome domains assessed: (i) anthropometric outcomes related to obesity (e.g., BMI, BMI z-score, body weight, waist circumference), (ii) physical activity outcomes, and (iii) nutrition-related and other health-related behavioural outcomes.

The methodological quality of the included studies was subsequently assessed using the Effective Public Health Practice Project (EPHPP) Quality Assessment Tool [17]. Each EPHPP domain was rated as strong, moderate, or weak according to the criteria specified in the EPHPP Quality Assessment Tool manual (Table 1). For the study design domain, randomized controlled trials and cluster-randomized controlled trials were generally rated as strong, controlled clinical trials and quasi-experimental studies as moderate, and weaker non-experimental designs as weak. Similar criteria were applied across the remaining domains, including selection bias, confounders, blinding, data collection methods, and withdrawals/dropouts. The overall methodological quality rating was then determined according to the EPHPP guidelines: studies with no weak ratings were classified as strong, studies with one weak rating as moderate, and studies with two or more weak ratings as weak.

## 3. Results

### 3.1. Study Selection and Characteristics

**Figure 1:**
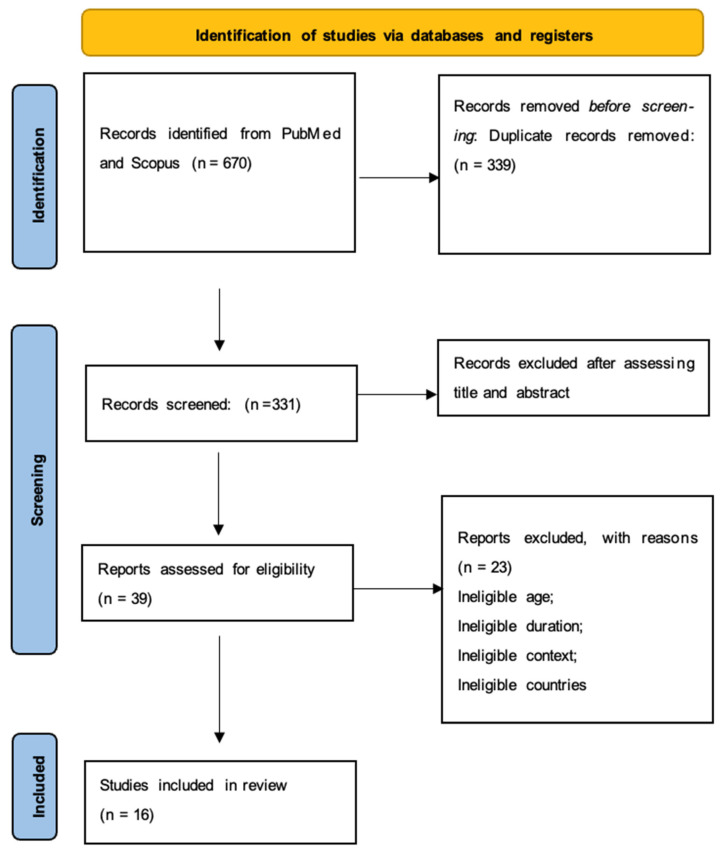
Flow diagram of studies through the review process [16].

In the first screening based on databases, 670 studies (PubMed: 356, Scopus: 314) were obtained. Having removed the duplicated studies (339), 331 remained and were read and evaluated by the researcher. After the title and abstract review (292 articles were excluded), the full texts of 39 studies in the databases were assessed. Following full-text evaluation, 16 studies met the inclusion criteria and were included in the final analysis. The study selection process is summarized in the PRISMA flow diagram (Figure 1).

### 3.2. Study Setting, Design and Population

Sixteen studies that were carried out across nine European countries (Ireland, Norway, Poland, Italy, England, Spain, Netherlands, Germany, Switzerland) met the eligibility criteria (Table 2). Most studies were randomized controlled trials (75%), while 12.5% employed a quasi-experimental design, and 6.25% each were classified as a retrospective cohort study and a clinical trial.

The duration of the interventions ranged from nine months [33] to five years [22]. Most of the interventions were conducted in primary and secondary school were 81.25% and 18.75%, respectively.

### 3.3. Quality of the Selected Studies

As shown in Table 3, the methodological quality of the included studies was generally high. Seven studies (43.75%) were rated as having strong methodological quality [18,20,21,22,24,25,27], six studies (37.5%) were classified as moderate quality [23,26,29,30,31,32], and three studies (18.75%) were rated as weak quality [19,28,33].

Weak methodological ratings were mainly attributable to insufficient reporting of blinding procedures, withdrawals and dropouts, and, in some cases, data collection methods. Given the predominance of studies rated as strong or moderate quality (81.25%), the overall findings of this review are supported by evidence of generally good methodological quality. Nevertheless, findings from studies classified as weak quality were interpreted with caution when synthesizing results and drawing conclusions.

The detailed methodological characteristics of all included studies are presented in Table 3.

### 3.4. Anthropometric Outcomes Related to Obesity

Fourteen of the sixteen included studies assessed obesity-related anthropometric outcomes, including BMI, BMI z-score, waist circumference, waist-to-height ratio, body fat percentage, body composition, or overweight/obesity prevalence. Positive outcomes were defined as statistically significant improvements in at least one obesity-related anthropometric measure compared with baseline values and/or control groups according to the original study analyses.

Among these fourteen studies, eleven (78.6%) reported statistically significant improvements in at least one anthropometric outcome [19,20,21,22,24,25,26,28,29,31,33], whereas three studies [18,27,30] found no significant effect on obesity-related measures.

Intervention duration among studies reporting favorable anthropometric outcomes ranged from nine months to four years, with most effective interventions lasting at least one academic year. Several successful interventions adopted multicomponent approaches combining physical activity promotion, nutrition education, environmental modifications, and parental involvement. Examples include the EAT Project [19], Project Spraoi [28], the DOiT intervention [31], and the EdAl study [26].

The effectiveness of interventions was not uniform across all population groups. In the HEIA study [21], significant improvements in BMI and BMI z-score were observed among girls but not boys, and beneficial effects were more pronounced among children from families with higher parental educational attainment. Similarly, the EdAl study [26] reported reductions in BMI z-scores primarily among girls. These findings suggest that participant characteristics, including sex and family educational background, may influence intervention effectiveness.

Among studies reporting no significant anthropometric improvements, common challenges included incomplete implementation of intervention components [18], variability in participant engagement, and difficulties in achieving sustained behavioural change [27,30] (Table 4).

### 3.5. Physical Activity Outcomes

Nine studies assessed physical activity outcomes using accelerometers, fitness tests, self-reported questionnaires, or combinations of these methods [19,23,24,26,29,30,32,33].

Evidence regarding physical activity outcomes was less consistent than for anthropometric measures. Improvements in physical activity levels or physical fitness were reported in several studies, particularly those incorporating structured physical education sessions, active breaks during school hours, extracurricular activities, and parental involvement [19,24,26]. The KISS study [24] demonstrated improvements in physical activity and fitness alongside reductions in adiposity, while the EdAl study [26] reported higher levels of after-school physical activity among intervention participants.

However, other studies found limited or no significant effects on physical activity behaviour [23,29,30], highlighting the challenges associated with achieving and maintaining behavioural change. Differences in measurement methods, intervention intensity, participant adherence, and follow-up duration may partly explain the heterogeneity of findings (Table 4).

### 3.6. Nutrition-Related and Other Behavioural Outcomes

Eight studies evaluated nutrition-related outcomes, including dietary habits, nutrition knowledge, sugar-sweetened beverage consumption, snack consumption, breakfast frequency, dietary quality, and fibre intake [19,25,26,28,31,33].

Overall, evidence regarding nutrition-related outcomes was mixed but generally favorable. Several studies reported significant improvements in nutrition knowledge and dietary behaviours following school-based interventions. Project Spraoi [28] demonstrated improvements in nutrition knowledge and dietary intake, while Wadolowska et al. [33] reported significant increases in nutrition knowledge following a school-based educational programme. Ermetici et al. [19] observed reductions in the consumption of sugar-sweetened beverages and high-energy snacks among adolescents receiving a multicomponent intervention.

The DOiT intervention [31] showed reductions in sugar-sweetened beverage consumption among girls and improvements in breakfast frequency among boys. Interventions that combined educational activities with environmental modifications and parental engagement appeared more likely to achieve favorable nutrition-related outcomes than educational approaches alone.

Taken together, these findings suggest that school-based interventions can positively influence dietary behaviours and nutrition knowledge, although effects vary according to intervention design, duration, implementation fidelity, and participant characteristics.

## 4. Discussion

The present systematic review aimed to summarize the available evidence on school-based interventions to prevent childhood overweight and obesity within the European context. Overall, 11 of the 14 studies that assessed BMI or anthropometric outcomes reported positive effects. However, evidence from several interventions suggests that these effects are not uniform across populations. In the HEIA study, Grydeland et al. [21] and Llauradó et al. [26] reported beneficial effects on BMI only among girls, and in the HEIA intervention, these effects were further restricted to girls from families with a higher educational background. These findings highlight the importance of considering participant characteristics, including sex and socioeconomic background, when designing and implementing childhood obesity prevention programmes.

A possible explanation for the lack of significant BMI effects observed in Adab et al. [18] and Lloyd et al. [27] may relate to limitations in intervention implementation and participant engagement. In the WAVES study [18], parental consent was obtained from only 60% of eligible children, and not all schools delivered every intervention component according to protocol. Similarly, the HeLP programme [27] encouraged children and families to identify behavioural changes that were most relevant to them. Several interventions included parental involvement as part of a broader multicomponent strategy. Although the evidence does not allow firm conclusions regarding the independent effect of parental engagement, family participation may support the adoption and maintenance of healthy lifestyle behaviours among children.

Another important component highlighted in the EdAl study [26] was parental involvement, as parents play a key role in shaping children’s dietary habits, physical activity patterns, and other health-related behaviours. Several of the interventions reporting favourable outcomes incorporated parental participation alongside educational, behavioural, and environmental strategies, suggesting that childhood obesity prevention may benefit from approaches extending beyond the school setting alone.

The evidence regarding physical activity outcomes was less consistent than that observed for anthropometric outcomes. While some interventions reported improvements in physical activity levels or physical fitness [19,24,26], others found limited or no significant effects [23,29,30]. This heterogeneity may reflect differences in intervention content, duration, intensity, implementation fidelity, and outcome assessment methods.

An additional consideration when interpreting the heterogeneous effects of physical activity interventions is the physiological impact of obesity itself on movement and exercise performance. Obesity is not only characterized by excess adiposity but is also associated with alterations in skeletal muscle structure and function, musculotendinous properties, movement efficiency, fatigue resistance, and exercise tolerance. These alterations may reduce the ability and motivation of children and adolescents with obesity to engage in physical activity and may therefore influence responsiveness to physical activity interventions. Previous research has shown that obesity can negatively affect muscle quality, tendon properties, and functional performance across the lifespan [34,35]. More recent evidence has also demonstrated that obesity is associated with impaired respiratory mechanics and reduced exercise efficiency, partly due to biomechanical and musculotendinous adaptations that increase the physiological cost of movement [36,37]. These mechanisms may help explain why improvements in physical activity behaviours are often less consistent than improvements in anthropometric outcomes and further support the need for long-term, multicomponent interventions that combine behavioural, educational, environmental, and family-based approaches. In addition, experimental evidence suggests that combined lifestyle strategies, including dietary modification and physical activity, may be necessary to effectively counteract obesity-related muscle and tendon dysfunction [38].

With regard to nutrition-related outcomes, several studies reported improvements in dietary behaviours and nutrition knowledge following school-based interventions. The most favourable findings were generally observed in multicomponent interventions combining educational activities with environmental modifications and parental involvement. Since changing behaviour is a complex and gradual process, improvements in nutrition knowledge may contribute to healthier dietary choices over time. Consistent with the findings of Project Spraoi [28] and the ABC of Healthy Eating study [33], several interventions reported significant improvements in nutrition knowledge and selected dietary behaviours.

The findings of this review also suggest that multicomponent interventions may be more effective than single-component approaches. Successful programmes commonly combined physical activity promotion, nutrition education, environmental modifications within the school setting, and family engagement. However, considerable heterogeneity existed across studies regarding intervention content, duration, implementation strategies, and outcome measures, making direct comparisons difficult.

The findings of this review are broadly consistent with previous systematic reviews reporting beneficial effects of school-based obesity prevention programmes. However, by focusing exclusively on European studies published between 2010 and 2026, the present review provides an updated regional perspective and highlights important differences between anthropometric and behavioural outcomes. In particular, while improvements in obesity-related anthropometric measures were frequently observed, evidence regarding physical activity and nutrition-related behaviours was more heterogeneous. The review also highlights the potential influence of intervention duration, multicomponent intervention design, participant characteristics, and methodological quality on intervention effectiveness.

This review has several limitations. First, although the review was conducted and reported according to the PRISMA guidelines, it was not prospectively registered, and no review protocol was developed prior to study selection. Consequently, methodological decisions were not documented in advance, which may reduce transparency and reproducibility. Second, the search strategy was limited to two electronic databases (PubMed and Scopus). Although these databases index a substantial proportion of the relevant literature, potentially eligible studies indexed exclusively in other databases, such as Web of Science, Embase, or the Cochrane Library, may have been missed. Furthermore, despite the use of multiple search terms related to physical activity, nutrition, and school-based programmes, the search strategy may not have captured the full spectrum of obesity prevention interventions implemented in school settings.

Third, the included studies varied substantially in terms of intervention characteristics, duration, outcome measures, and methods of assessment, precluding quantitative synthesis through meta-analysis. In addition, the studies included in this review used a variety of outcome assessment methods. While several studies assessed dietary behaviours and physical activity using self-reported questionnaires, others employed objective measures such as accelerometers, fitness tests, or direct anthropometric measurements. This methodological heterogeneity should be considered when interpreting the findings, as self-reported measures may be subject to recall and social desirability bias. Fourth, although the majority of studies were rated as having strong or moderate methodological quality, some studies presented limitations related to blinding procedures, reporting of withdrawals and dropouts, and data collection methods.

Finally, the review focused exclusively on studies conducted in European countries. While this approach allowed the examination of interventions within relatively comparable educational, social, and policy contexts, it may limit the generalizability of the findings to other regions. Moreover, the geographical distribution of included studies was uneven, with some European regions, particularly Eastern and lower-income countries, being underrepresented. Differences in school food policies, physical education curricula, socioeconomic conditions, and healthcare systems across European countries may also influence intervention effectiveness. Future research should therefore focus on generating evidence from underrepresented European settings and exploring how contextual factors shape the effectiveness and sustainability of school-based obesity prevention programmes.

Despite these limitations, the review provides an updated synthesis of European school-based interventions addressing childhood obesity and highlights the potential benefits of comprehensive, multicomponent approaches. Future research should focus on identifying which intervention components are most effective, how these effects vary across population groups, and how successful programmes can be sustainably implemented within diverse educational and social contexts.

## 5. Conclusions

The findings of this systematic review suggest that school-based interventions implemented in European countries can contribute to the prevention and reduction in childhood obesity, particularly when delivered as long-term, multicomponent programmes that combine physical activity promotion, nutrition education, environmental modifications, and parental engagement. Most studies assessing anthropometric outcomes reported favorable effects on measures such as BMI, BMI z-score, waist circumference, body composition, or overweight/obesity prevalence.

However, the evidence regarding physical activity, dietary behaviours, and other health-related behavioural outcomes was more heterogeneous. While several interventions reported improvements in nutrition knowledge, dietary habits, physical activity levels, or sedentary behaviours, these effects were not consistently observed across all studies. Differences in intervention characteristics, duration, implementation fidelity, participant characteristics, and outcome assessment methods may partly explain this variability.

Overall, the findings support the potential value of comprehensive school-based approaches for childhood obesity prevention. Nevertheless, further high-quality research is needed to better understand which intervention components are most effective, how behavioural changes can be sustained over time, and how intervention effectiveness may vary across different population groups and educational settings.

## Figures and Tables

**Table 1:** Quality assessment for studies [17].

Quality Assessment Tool for Quantitative Studies	
(A) Selection bias	Do the individuals selected for the study represent the target population? Number of individuals participating in the study.
(B) Study design	Is the study defined as randomized? If yes, is the randomization method specified? If yes, is the method appropriate?
(C) Confounders	Are there significant differences between pre-intervention groups? If yes, is the proportion of situations that cause confusion about the design or analysis indicated?
(D) Blinding	Are the assessors aware of the intervention or the exposure of the participants? Are the study participants aware of the research questions?
(E) Data collection methods	Is the validity of the data collection tools shown? Has the reliability of data collection tools been demonstrated?
(F) Withdrawals and dropouts	Were those who quit or were unable to complete the report? Is the proportion of those completing the study indicated?

**Table 2:** Summary of the selected studies.

Study	Design	Participants	Aims	Program’s Duration
Adab et al. (2018) [18] Effectiveness of a childhood obesity prevention programme delivered through schools, targeting 6- and 7-year-olds: cluster randomized controlled trial (WAVES study).	RCT	6–7 years old. 53 schools remained in the trial. 1287 (87.7%) and 1169 (79.7%) pupils were available at first follow-up (15 months) and second follow-up (30 months), respectively.	Assess the effectiveness of a school and family-based healthy lifestyle programme (WAVES intervention) compared with usual practice, in preventing childhood obesity.	1 year
Ermetici et al. (2016) [19] Association Between a School-Based Intervention and Adiposity Outcomes in Adolescents: The Italian “EAT” Project	Quasi-experimental study	11–15 years old 3 schools. 487 adolescents (IG: 262; CG: 225).	Aimed to evaluate whether a school-based multicomponent educational program of the duration of two school years could improve adiposity measures, mainly BMI z-score and WHtR, in middle-school adolescents.	2 years
Głąbska et al. (2019) [20] The National After-School Athletics Program Participation as a Tool to Reduce the Risk of Obesity in Adolescents after One Year of Intervention: A Nationwide Study	RCT	1014 adolescents aged 12–13: 507 individuals for the Athletics All program (210 boys, 297 girls) and 507 pair-matched individuals not participating in any physical activity program (matching including: gender, age, city of residence).	Assess the influence of participation in a national after-school athletics program (conducted in school settings, three sessions a week, ensuring regular participation and including nutritional education of trainers) on the risk of obesity and body composition in a nationwide sample of boys and girls after one year of intervention.	1 year
Grydeland et al. (2013) [21] Effects of a 20-month cluster randomized controlled school-based intervention trial on BMI of school-aged boys and girls: the HEIA study	RCT	11 years old 12 schools (IG: 784 children) and 25 schools to the CG: 1381 children).	Investigate the effects of a systematically developed, 20-month multicomponent school-based intervention programme, the HEIA study, on BMI. The effect of intervention on anthropometry was influenced by gender, pubertal status, or level of parental education.	20 months
Jansen et al. (2011) [22] Effectiveness of a primary school-based intervention to reduce overweight	RCT	6–12 years old 20 schools 2622 children	To evaluate the effect of a school-based intervention program to reduce overweight and improve fitness in primary school children.	1 academic year
Kipping et al. (2014) [23] Effect of intervention aimed at increasing physical activity, reducing sedentary behavior, and increasing fruit and vegetable consumption in children: Active for Life Year 5 (AFLY5) school-based cluster randomize controlled trial	RCT	8–9 years old 60 primary schools in the southwest of England. 2221 Primary school children (IG: 1064, CG: 1157).	To investigate the effectiveness of a school-based intervention to increase PA, reduce sedentary behavior, and increase fruit and vegetable consumption in children.	5 years
Kriemler et al. (2010) [24] Effect of school based physical activity programme (KISS) on fitness and adiposity in primary schoolchildren: cluster randomized controlled trial	RCT	28 classes from 15 elementary schools in Switzerland. 502 children (IG: 297, CG: 205). First and fifth grade.	To assess the effectiveness of a school-based PA program during one school year on physical and psychological health in children.	1 academic year
Llargués et al. (2012) [25] Medium-term evaluation of an educational intervention on dietary and physical exercise habits in schoolchildren: The Avall 2 study	RCT	First year schoolchildren. (IG: 272; CG: 337).	To assess whether the benefits seen in nutrition, physical activity, and body mass index were maintained at 2 years of completion of the educational intervention.	2 years
Llauradó et al. (2018) [26] Follow-up of a healthy lifestyle education program (the EdAl study): four years after cessation of randomized controlled trial intervention	RCT	13–15 years old. (IG: 349; CG: 154).	To assess the sustainability of benefits four years after the cessation of the post-Educació en Alimentació (EDAl) program intervention by evaluating obesity-related outcomes and lifestyle behaviors in adolescents aged 13 to 15 years.	4 years
Lloyd et al. (2018) [27] Effectiveness of the Healthy Lifestyles Programme (HeLP) to prevent obesity in UK primary-school children: a cluster randomized controlled trial	RCT	9–10-year-old 16 schools (676 children) were assigned to the intervention group and 16 schools (648 children) to the control group.	Establish whether a school-based intervention for children aged 9–10 years would prevent excessive weight gain after 24 months.	2 years
Merrotsy et al. (2019) [28] Project Spraoi: a two-year longitudinal study on the effectiveness of a school-based nutrition and physical activity intervention on dietary intake, nutritional knowledge and markers of health of Irish schoolchildren	RCT	Two primary schools (one intervention and one control) in Cork, Ireland. Participants included six-year-old children (*n* = 49; mean age = 6.09 years) and ten-year-old children (*n* = 52; mean age = 9.90 ± 0.37 years).	To assess the effectiveness of nutrition and physical activity (PA) intervention on dietary intake (DI), nutritional knowledge (NK), blood pressure (BP), anthropometric measures, and cardiorespiratory fitness (CRF) of schoolchildren.	2 academic years
Siegrist et al. (2013) [29] Effects of a physical education program on physical activity, fitness, and health in children: The JuvenTUM project	RCT	8-year-old children 4 intervention schools 4 control schools *N* = 826 (IG: 427; CG: 297)	To increase physical activity, fitness, and lifestyle awareness, and to improve health obesity measures.	1 year
Smit et al. (2025) [30] Long-term effects of a primary school-based overweight preventive intervention on physical fitness and physical activity: a propensity score-matched retrospective cohort study within the Generation R Study	Retrospective Cohort	4129 adolescents from the Generation R cohort with linked school data (subsamples: fitness *n*: 1826; physical activity *n*: 1258).	To evaluate long-term effects of the Lekker Fit! primary school-based overweight prevention programme on physical activity, fitness and weight-related outcomes.	≥1 academic year with extended follow-up
van Nassau et al. (2014) [31] The Dutch Obesity Intervention in Teenagers (DOiT) cluster-controlled implementation trial: intervention effects and mediators and moderators of adiposity and energy balance-related behaviors	RCT	12–14 years old 1486 adolescents (CG: 484; IG: 1002) were included in the analysis (questionnaire data and at least one measure of adiposity provided at both time points).	To evaluate the intervention effects of the DOiT-implementation programme on adolescents’ adiposity and EBRBs during natural dissemination; to test the EBRB mediating factors underlying the DOiT implementation intervention effects on adolescents’ adiposity; and to explore whether gender, ethnicity and ad	20 months
Verjans-Janssen et al. (2020) [32] Effects of the KEIGAAF intervention on the BMI z-score and energy balance-related behaviors of primary school-aged children	Quasi- Experimental study	7–10 years old (mean age = 8.5) 54% Girls. 523 children (CG and IG combined).	To determine the sustainability of diet related and lifestyle-related school-based education on sedentary and active lifestyle, diet quality and body composition of Polish pre-teenagers in a medium-term follow-up study.	1–2 academic years
Wadolowska et al. (2019) [33] Changes in Sedentary and Active Lifestyle, Diet Quality and Body Composition Nine Months after an Education Program in Polish Students Aged 11–12 Years: Report from the ABC of Healthy Eating Study	Clinical Trial	11–12 years old.464 students (CG: 145; IG: 319).	To determine the sustainability of diet related and lifestyle-related school-based education on sedentary and active lifestyle, diet quality and body composition of Polish pre-teenagers in a medium-term follow-up study.	9 months

Note: “IG” was used for intervention groups and “CG” control groups. RCT was used for Randomized Clinical Trial.

**Table 3:** Quality assessment based on Thomas et al. [17].

Authors	Selection Bias	Study Design	Confounders	Blinding	Data Collection Methods	Withdrawals and Dropouts	Overall Score
Adab et al. (2018) [18]	Strong	Strong	Strong	Strong	Strong	Strong	Strong
Ermetici et al. (2016) [19]	Strong	Weak	Strong	Weak	Moderate	Weak	Weak
Głąbska et al. (2019) [20]	Strong	Strong	Strong	Moderate	Strong	Strong	Strong
Grydeland et al. (2013) [21]	Strong	Strong	Strong	Moderate	Moderate	Strong	Strong
Jansen et al. (2011) [22]	Strong	Strong	Strong	Moderate	Strong	Strong	Strong
Kipping et al. (2014) [23]	Strong	Strong	Strong	Strong	Moderate	Weak	Moderate
Kriemler et al. (2010) [24]	Strong	Strong	Strong	Moderate	Strong	Strong	Strong
Llargués et al. (2012) [25]	Strong	Strong	Strong	Moderate	Strong	Strong	Strong
Llauradó et al. (2018) [26]	Weak	Strong	Strong	Moderate	Moderate	Strong	Moderate
Lloyd et al. (2018) [27]	Strong	Strong	Strong	Strong	Strong	Strong	Strong
Merrotsy et al. (2019) [28]	Strong	Strong	Strong	Weak	Strong	Weak	Weak
Siegrist et al. (2013) [29]	Strong	Strong	Strong	Moderate	Weak	Strong	Moderate
Smit et al. (2025) [30]	Moderate	Moderate	Strong	Weak	Strong	Moderate	Moderate
van Nassau et al. (2014) [31]	Strong	Strong	Strong	Moderate	Strong	Moderate	Moderate
Verjans-Janssen et al. (2020) [32]	Moderate	Moderate	Strong	Weak	Moderate	Moderate	Moderate
Wadolowska et al. [33]	Strong	Moderate	Strong	Weak	Moderate	Weak	Weak

**Table 4:** Characteristics and Outcomes of School-Based Interventions for the Prevention and Management of Childhood Obesity.

Study	Intervention Type	Duration	Primary Outcomes	Key Findings
Adab et al. (2018) [18]	Multicomponent intervention including additional physical activity, healthy eating education, and parental cooking workshops (WAVES)	12 months	BMI z-score, adiposity, physical activity, dietary behaviours	No significant effect on BMI z-score or obesity prevention.
Ermetici et al. (2016) [19]	School environmental modifications, nutrition education, pedometers, and parental involvement (EAT Project)	12 months	BMI z-score, WHtR, physical activity, dietary behaviours	Significant improvements in BMI z-score and WHtR; increased physical activity and healthier dietary behaviours.
Głąbska et al. (2019) [20]	After-school athletics programme combined with nutrition education	12 months	BMI percentile, body composition, waist circumference	Reduced BMI percentile, abdominal obesity, and fat mass; increased muscle mass.
Grydeland et al. (2013) [21]	Multicomponent physical activity intervention with classroom activities, equipment provision, and parental engagement (HEIA)	20 months	BMI, BMI z-score, waist circumference	Improvements in BMI and BMI z-score among girls; no significant effects on waist circumference.
Jansen et al. (2011) [22]	Physical education, classroom education, sports activities, and parental involvement	24 months	BMI, overweight prevalence, waist circumference, fitness	Reduced overweight prevalence and waist circumference; improved physical fitness.
Kipping et al. (2014) [23]	Healthy lifestyle programme including physical activity, dietary education, teacher training, and parental activities (AFLY5)	12 months	Physical activity, sedentary behaviour, BMI, dietary behaviours	No significant improvement in physical activity; favourable effects on BMI and waist circumference.
Kriemler et al. (2010) [24]	Enhanced physical education, activity breaks, and physical activity homework (KISS)	9 months	Physical activity, fitness, adiposity	Improved physical activity and fitness; reduced adiposity.
Llargués et al. (2012) [25]	Educational intervention promoting healthy diet and physical activity (Avall 2)	24 months	BMI, overweight/obesity prevalence, lifestyle behaviours	Lower increase in BMI and obesity prevalence compared with controls.
Llauradó et al. (2018) [26]	Healthy lifestyle education programme with parental participation (EdAl)	4-year follow-up	BMI z-score, obesity prevalence, physical activity, dietary behaviours	Reduced BMI z-scores among girls; lower obesity prevalence among boys; increased after-school physical activity.
Lloyd et al. (2018) [27]	HeLP programme including education, physical activity workshops, drama activities, and parental support	24 months	BMI, physical activity, dietary behaviours	No significant effect on overweight or obesity prevention.
Merrotsy et al. (2019) [28]	Project Spraoi: nutrition education, physical activity sessions, and parental engagement	24 months	WHtR, blood pressure, nutrition knowledge, dietary behaviours	Improved WHtR, blood pressure, nutrition knowledge, and selected dietary outcomes.
Siegrist et al. (2013) [29]	Physical education and health promotion programme (JuvenTUM)	12 months	Physical activity, fitness, waist circumference	Reduced waist circumference; no significant intervention effects on physical activity.
Smit et al. (2025) [30]	Multicomponent programme including physical activity promotion, nutrition education, environmental modifications, and parental engagement	Long-term follow-up	Physical fitness, physical activity	No favourable long-term effects on physical fitness or physical activity.
van Nassau et al. (2014) [31]	DOiT intervention targeting energy balance-related behaviours	24 months	BMI z-score, dietary behaviours, physical activity behaviours	No overall intervention effects; subgroup improvements in breakfast frequency and sugar-sweetened beverage consumption.
Verjans-Janssen et al. (2020) [32]	KEIGAAF multicomponent school-based intervention	24 months	BMI z-score, physical activity, sedentary behaviour	Improved physical activity-related behaviours and reduced sedentary behaviour; limited effects on BMI z-score.
Wadolowska et al. (2019) [33]	ABC of Healthy Eating educational programme	9-month follow-up	Nutrition knowledge, diet quality, waist circumference, physical activity	Improved nutrition knowledge and waist-related outcomes; mixed effects on physical activity.

## Data Availability

No new data were created or analyzed in this study. Data sharing is not applicable to this article.

## Abbreviations

The following abbreviations are used in this manuscript:

WHO	World Health Organization
BMI	Body Mass Index
BMIz	Body Mass Index z-score
PA	Physical Activity
PE	Physical Education
MVPA	Moderate-to-Vigorous Physical Activity
PRISMA	Preferred Reporting Items for Systematic Reviews and Meta-Analyses
EPHPP	Effective Public Health Practice Project
RCT	Randomized Controlled Trial
IG	Intervention Group
CG	Control Group
IOTF	International Obesity Task Force
WC	Waist Circumference
WHtR	Waist-to-Height Ratio
BP	Blood Pressure
BMR	Basal Metabolic Rate
BIA	Bioelectrical Impedance Analysis
OR	Odds Ratio
CI	Confidence Interval
ES	Effect Size
CDC	Centers for Disease Control and Prevention
CADET	Child and Diet Evaluation Tool
CBIS	Child’s Body Image Scale
PedsQL	Pediatric Quality of Life Inventory
FIQ	Food Intake Questionnaire
IVAC	Investigation, Vision, Action and Change
EBRB	Energy Balance-Related Behaviour
WAVES	West Midlands ActiVe lifestyle and healthy Eating in School children Study
EAT	Educazione Alimentare Teenagers
HEIA	Health In Adolescents
AFLY5	Active for Life Year 5
KISS	Kinder-Sportstudie
EdAl	Educació en Alimentació
HeLP	Healthy Lifestyles Programme
DOiT	Dutch Obesity Intervention in Teenagers
KEIGAAF	Krachtige Eerstelijnszorg voor Kinderen en Gezinnen: Aanpak Activiteiten en Fitter worden
NK	Nutrition Knowledge
SF-FFQ4.PolishChildren	Short Form Food Frequency Questionnaire for Polish Children
nHDI	Non-Healthy Diet Index
